# Combined Analysis of DNA Methylome and Transcriptome Reveal Novel Candidate Genes Related to Porcine *Escherichia coli* F4ab/ac-Induced Diarrhea

**DOI:** 10.3389/fcimb.2020.00250

**Published:** 2020-05-29

**Authors:** Wenwen Wang, Chuanli Zhou, Hui Tang, Ying Yu, Qin Zhang

**Affiliations:** ^1^Shandong Provincial Key Laboratory of Animal Biotechnology and Disease Control and Prevention, Shandong Agricultural University, Taian, China; ^2^College of Animal Science and Technology, China Agricultural University, Beijing, China

**Keywords:** DNA methylome, transcriptome, enterotoxigenic *Escherichia coli*, diarrhea, pig

## Abstract

Enterotoxigenic *Escherichia coli* (ETEC) that express F4 (K88) fimbriae are the principal microorganisms responsible for bacterial diarrhea in neonatal and pre-weaning piglets. To better understand the molecular effects of ETEC F4ab/ac infection, we performed a genome-wide comparison of the changes in DNA methylation and gene expression in ETEC F4ab/ac infected porcine intestinal epithelial cells. We characterized the pattern of changes in methylation and found 3297 and 1593 differentially methylated regions in cells infected with F4ab and F4ac, respectively. Moreover, 606 and 780 differentially expressed genes (DEGs) in ETEC F4ab and F4ac infected cells were detected and these genes were highly enriched in immune/defense response related pathways. Integrative analysis identified 27 and 10 genes showing inverse correlations between promoter methylation and expression with ETEC F4ab/ac infection. Altered DNA methylation and expression of various genes suggested their roles and potential functional interactions upon ETEC F4ab/ac infection. Further functional analyses revealed that three DEGs (*S100A9, SGO1*, and *ESPL1*) in F4ab infected cells and three DEGs (*MAP3K21, PAK6*, and *MPZL1*) in F4ac infected cells are likely involved in the host cells response to ETEC infection. Our data provides further insight into the epigenetic and transcriptomic alterations of ETEC F4ab/ac infected porcine intestinal epithelial cells, and may advance the identification of biomarkers and drug targets for predicting susceptibility to and controlling ETEC F4ab/ac induced diarrhea.

## Introduction

Enterotoxigenic *Escherichia coli* (ETEC) with F4 (K88) fimbriae is the leading cause of diarrhea in neonatal and pre-weaning piglets, resulting in levels of illness and mortality that have become a major economic burden to the pig farming industry worldwide (Wang W. et al., 2019). Three variants of the F4 strain, F4ab, F4ac, and F4ad, can be distinguished serologically (Li et al., 2007). The “a” is a common antigenic factor, whereas “b”, “c”, and “d” represent specific epitopes (Sinha et al., 2019). The fimbriae of these three variants share similarities in their structures including the major subunit, FaeG, and several minor subunits (FaeF, FaeH, FaeC, probably FaeI, and FaeJ), all of which are controlled by a single gene cluster (Xia et al., 2015). Of these three variants, F4ab and F4ac are most commonly associated with ETEC-induced diarrhea (Nguyen et al., 2017). Comparative analysis of the sequences of the F4ab and F4ac genes revealed that the differences between these two serotypes are confined to the *faeG* gene, which differs in amino acid composition; different localizations of “b” and “c” epitope; and different specificities in attachment to receptors (Van den Broeck et al., 2000). Identifying control strategies for ETEC F4ab/ac-induced piglet diarrhea is highly important for promoting the development of swine industry worldwide.

DNA methylation is one of the central epigenetic modifications; in mammalian genomes it occurs mainly on cytosines at position C5 in CpG dinucleotides (Wang H. et al., 2019). DNA methylation is involved in numerous processes, such as genomic imprinting, transcriptional regulation, and tumorigenesis (Schuebeler, 2015), and it occurs in response to environmental factors, such as pathogen stimulation, drug treatment, pollutants, and disease, and it serves to regulate expression of the responsive genes (Kiga et al., 2014; Jiang et al., 2018; Swathy et al., 2018; Chen et al., 2019). Bacterial endotoxins have profound impacts on gene expression in intestinal epithelial cells through DNA methylation modifications. The expression of *FUT1* (Dai et al., 2017) and *FUT2* (Wu et al., 2018) are epigenetically modulated by DNA methylation of their promoters, regulating ETEC F18 resistance in weaned piglets. Systematic investigations on the global DNA methylation changes induced by ETEC F4ab/ac infection and the methylation pattern of responsive genes are still scant.

This study aimed to determine the distribution of methylation on the DNA in porcine small intestine epithelial cells infected by ETEC F4ab/ac and to analyze potential DNA methylation targets related to the host cells' response to infection. A subset of DNA methylation target genes that were strongly correlated with susceptibility of ETEC F4ab/ac infection in piglets were identified. Our results enhance the understanding of epigenetic changes in intestinal cells in response to ETEC F4ab/ac infection, and may contribute to the identification of biomarkers and drug targets for predicting susceptibility to and controlling ETEC F4ab/ac induced diarrhea.

## Results

### Genome-Wide Methylation Profiles in ETEC F4ab/ac Infected IPEC-J2 Cells

**Figure 1:**
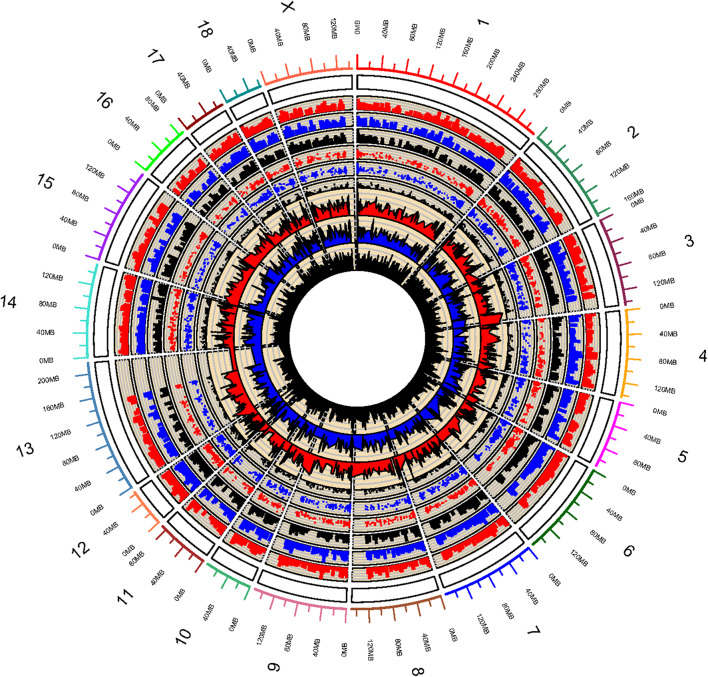
Global methylation pattern of IPEC-J2 cells infected with ETEC F4ab (the inner three tracks), ETEC F4ac (track 4, 5, 6 from inside to outside), and uninfected controls (track 7, 8, 9 from inside to outside). The numbers on the outermost track indicate the chromosome of the porcine genome.

Whole genome DNA methylation of triplicate samples of IPEC-J2 cells infected with ETEC F4ab, F4ac, and uninfected, were analyzed to determine methylation profiles of ETEC infection. Using a sliding-window peak-finding algorithm provided by NimbleScan v2.6 (Roche-NimbleGen), a total of 46,940 methylated enrichment peaks (EPs) were identified from the nine samples, of which 14,805 (31.54%) were in the ETEC F4ab infected samples, 16,336 (34.80%) in the ETEC F4ac infected samples, and 15,799 (33.66%) in the uninfected control samples (Table S1). As shown in the methylation map (Figure 1), while most chromosomal regions were covered by methylated peaks, the methylation density in these regions were distinct among the chromosomes; chromosome 13 in particular, contained a relatively large unmethylated region.

### Methylation Status in Genome CGIs of Infected and Uninfected IPEC-J2 Cells

**Figure 2:**
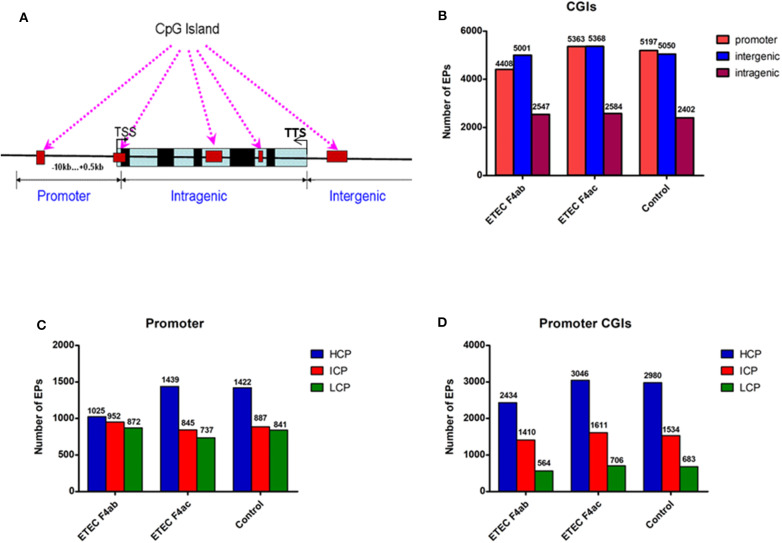
Distribution of DNA methylation enrichment peaks in IPEC-J2 cells infected with ETEC F4ab, ETEC F4ac, and uninfected. **(A)** Generic diagram showing CpG islands (CGIs) relative to gene transcript regions. TSS: transcriptional start site. **(B)** Number of methylation enrichment peaks (EPs) in each CGI region per experimental group. **(C)** Number of EPs in each type of promoter. HCP, high CpG density promoter; ICP, intermediate CpG density promoter; LCP, low CpG density promoter. **(D)** Number of EPs in the promoter CGIs.

In methylome studies, CpG islands (CGIs) are of particular interest because of their role in controlling gene expression (Jones, 2012). Therefore, we analyzed the methylation status of CGIs in the genome of the porcine intestinal epithelial cell line IPEC-J2 after infection with ETEC F4ab and F4ac. We grouped the CGIs into three classes according to their distance to the RefSeq genes: promoter CGIs [from about−10 kb to + 0.5 kb around the transcription start site (TSS)], intragenic CGIs [from + 0.5 kb around the TSS to the transcription terminal site (TTS)], and intergenic CGIs (those that do not fall into neither the promoter northe intragenic group) (Yu et al., 2014; Song et al., 2016; Figure 2A). The numbers of methylated EPs in the three classes of CGIs among the three groups of IPEC-J2 cells are shown in Figure 2B. Most of the methylated EPs were distributed in the intergenic and promoter CGIs for all three IPEC-J2 cell groups. It is worth mentioning that the ETEC F4ab-infected cells had relatively lower methylation levels in promoter CGIs than the uninfected or the ETEC F4ac-infected cells. The ETEC F4ac-infected cells had slightly higher methylation levels in promoter and intergenic CGIs than the uninfected or the ETEC F4ab-infected cells. In addition, the number of methylation EPs within intragenic CGIs in ETEC F4ab/ac-infected cells was higher than in uninfected cells.

Promoters are the main targets of DNA methylation modification, and the center to turn on or off gene expression (Wang X. et al., 2019). The key promoter region is defined as the −800 to +200 bp around the TSS. Based on the CpG ratio, GC content, and length CpG-rich region, we divided gene promoters into three types: high CpG density promoter (HCP), low CpG density promoter (LCP), and intermediate CpG density promoter (ICP) (Yu et al., 2014). We then analyzed the distribution of methylated EPs in the three types of the promoter (Figure 2C). We found that the number of methylated EPs was highest in the HCPs of all IPEC-J2 cell groups, followed by that in ICPs and LCPs. Additionally, in promoter CGIs, HCPs had more methylated EPs in all IPEC-J2 cell groups than ICPs or LCPs (Figure 2D). The activity of HCPs is negatively correlated with their DNA methylation status (Weber et al., 2007). We found that compared to the uninfected cells, the ETEC F4ab infected cells had fewer methylated EPs in HCPs, while the ETEC F4ac infected cells had slightly more methylated EPs in HCPs, this held true for promoter CGIs as well (Figures 2C,D).

**Figure 3:**
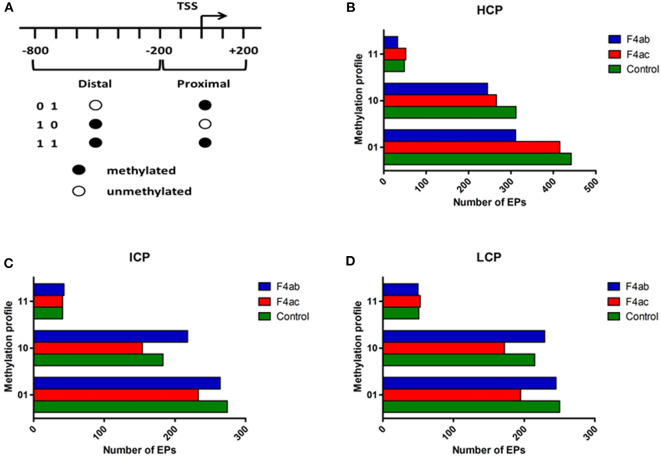
Distribution of different methylation patterns of promoter regions around the TSS in the three groups. **(A)** Schematic of the designated promoter regions and their methylation profiles. “0” denotes unmethylated and “1” methylated. The number of methylation peaks and the methylation profiles in **(B)** HCP, **(C)** ICP, and **(D)** LCP for each experimental group. 01: proximally methylated; 10: distally methylated; 11: fully methylated.

To more finely map the pattern of DNA methylation, we divided each type of promoter (HCP, ICP, LCP) into distal (−800 to −200bp) and proximal regions (−200 to +200bp) relative to the TSS. Each region was then defined as methylated (indicated by “1”) or unmethylated (‘0'). Thus, each type of promoter was classified into three subtypes according to their methylation profile (Figure 3A) (Koga et al., 2009). For each type of promoter, the methylation profile and the number of methylated EPs for each IPEC-J2 cell group are graphed in Figures 3B–D. In general, the promoters were either highly distally methylated (“10” pattern) or highly proximally methylated (“01”), but not both (“11”). Infection with ETEC F4ab and F4ac resulted in heterogeneous methylation patterns. For all promoter types, ETEC F4ac infected cells were hypomethylated with ‘01' and “10” patterns compared to uninfected cells. ETEC F4ab infected cells were hypomethylated for the HCP type in all patterns (Figure 3B). For the ICP and LCP types, F4ab infected cells were hypermethylated in the “10” pattern and hypomethylated in the “01” pattern. In the “11” pattern, ICPs, in F4ab infected cells were hypermethylated, and for LCPs they were hypomethylated (Figures 3C,D).

The DNA methylation changes and differences presumably reflect the ability of these pathogens to trigger epigenetic responses in cells involved in the immune system (Tarakhovsky, 2010).

### Identification of Differentially Methylated Genes

**Figure 4:**
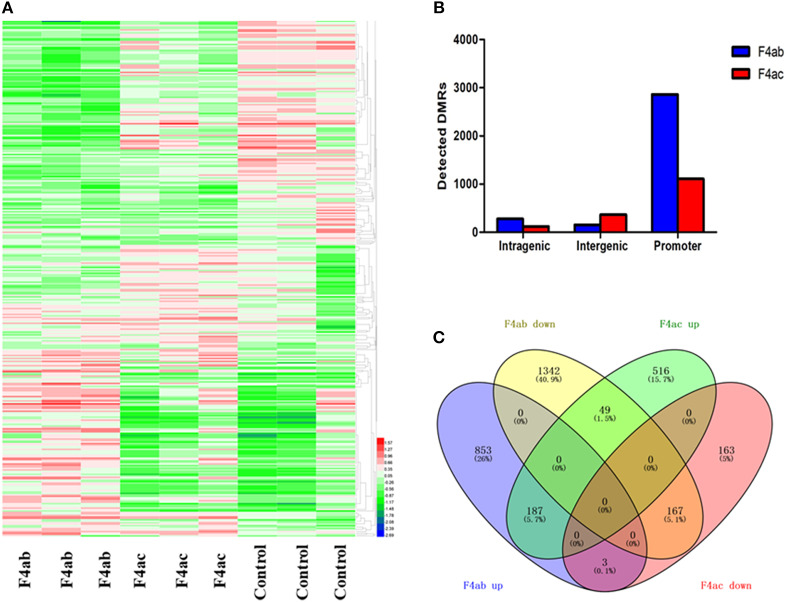
**(A)** Heatmap of differential methylation among the nine samples. F4ab: IPEC-J2 cells infected with F4ab ETEC; F4ac: IPEC-J2 cells infected with F4ac ETEC; Control: uninfected IPEC-J2 cells. **(B)** Distribution of DMRs. Genomic coordinates for these feature types were obtained from the UCSC pig reference genome. F4ab: DMRs between F4ab infected and uninfected cells; F4ac: DMRs between F4ac infected and uninfected cells. **(C)** Venn diagram showing the relationships among the differentially methylated genes identified. F4ab down/up: hypomethylated/hypermethylated genes between F4ab and uninfected cells; F4ac down/up: hypomethylated/hypermethylated genes between F4ac infected and uninfected cells.

To detect changes in the DNA methylome induced by ETEC F4ab/ac infection, we compared ETEC F4ab/ac infected and uninfected cells to identify differentially methylated regions (DMRs) in genomic DNA. 3297 DMRs were identified between ETEC F4ab infected and uninfected cells, of which 1261 were hypermethylated and 2036 hypomethylated in the infected cells (Table S2). 1593 DMRs were identified between ETEC F4ac infected and uninfected cells, of which 1140 were hypermethylated and 453 were hypomethylated in the infected cells (Table S3). The DNA methylation levels in the DMRs and the differences among the nine samples are shown by using a heatmap in Figure 4A. We subsequently mapped all DMRs to their nearest genomic features and found that DMRs were unevenly distributed across the genome. The majority of the DMRs were in the promoter regions in cells infected with ETEC F4ab or F4ac (Figure 4B), which constitute a large proportion in MeDIP-chip. To predict the potential functional significance of the identified DMRs, we analyzed the DMRs located in intragenic and promoter regions. The DMRs in the F4ab infected cells involve 2601 genes, of which, 2433 were differentially methylated only in promoters, 147 only in gene-bodies, and 21 in both promoters and gene-bodies (Table S4). Of the 2601 genes, 1043 were hypermethylated and 1558 hypomethylated. The DMRs in F4ac infected cells involve 1085 genes, of which, 991 were differentially methylated only in promoters, 91 only in gene-bodies, and 3 in both promoters and gene-bodies (Table S5). Of these 1085 genes, 752 were hypermethylated and 333 hypomethylated. Furthermore, 406 differentially methylated genes were common to both F4ab and F4ac infected cells. Of these, 187 were commonly hypermethylated and 167 commonly hypomethylated, and 52 hyper- or hypo-methylated in opposite directions in F4ab vs. F4ac infected cells (Figure 4C). Some of the identified genes involved in the immune system have been well-studied. For example, PUMA [*Bbc3*; p53 upregulated modulator of apoptosis (Okuda et al., 2017)] interacts with antiapoptotic Bcl-2 family members, leading to the formation of the free-type Bax and/or Bak, which are then able to signal apoptosis to the mitochondria (Yu and Zhang, 2008). RasGrf1 participates in the Ras signaling pathway (Manyes et al., 2014). JAK2 is in many ways the prototypical member of the JAK family, with an essential signaling role for cytokines and interferons involved in immunity and antiviral responses (Ferrao et al., 2018).

### Validation of MeDIP-Chip Data by Bisulfite Sequencing

**Figure 5:**
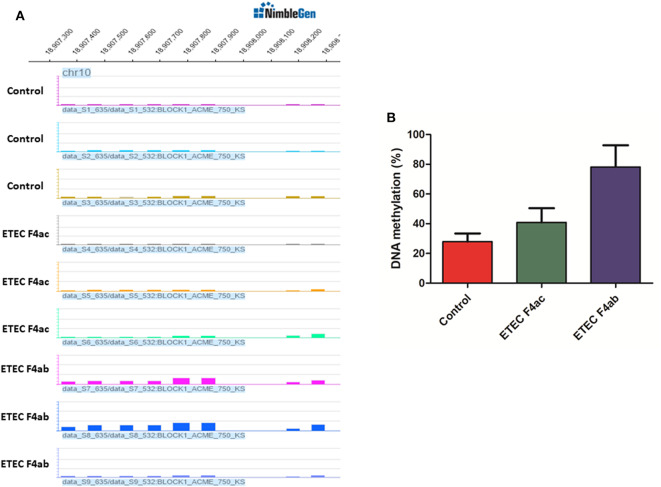
**(A)** Differentially methylated promoters of *TLR5* gene in untreated IPEC-J2 cells, ETEC F4ac infected cells, and ETEC F4ab infected cells by MeDIP-chip. **(B)** Validation of promoter methylation status through bisulfite sequencing PCR.

To assess the accuracy of the MeDIP-chip results, the *TLR5* gene was selected to validate promoter DNA methylation enrichment using bisulfite sequencing. The *TLR5* gene was chosen because results from MeDIP-chip data indicated that it was methylated at low levels in untreated IPEC-J2 cells, moderately methylated in ETEC F4ac infected cells, and highly methylated in ETEC F4ab infected cells (Figure 5A). The results from bisulfite sequencing showed that *TLR5* exhibited hypomethylated, moderate methylated, and hypermethylated enrichment in untreated IPEC-J2 cells, ETEC F4ac infected cells, and ETEC F4ab infected cells, respectively (Figure 5B), which are in good agreement with the results from the MeDIP-chip.

### Characterization of Transcriptomic Changes Induced by ETEC F4ab/ac Infection

We used an Agilent Porcine Oligo Microarray (4 × 44K) to analyze global gene expression in infected and uninfected cells. Genes were classified as differentially expressed if they exhibited |FC (fold-change)| > 1.5 with *q* < 0.05. In the ETEC F4ab infected cells we identified 606 genes that were significantly differentially expressed compared to the uninfected controls, 421 of these were up-regulated and 185 down-regulated (Table S6). In the ETEC F4ac infected cells, we identified 780 genes that were significantly differentially expressed compared to the uninfected controls, 524 of these were up-regulated and 256 down-regulated (Table S7). This genome-wide expression analysis provided a comprehensive portrait of the immune response, at the transcriptional level, of IPEC-J2 cells infected with ETEC F4ab/ac infection.

**Figure 6:**
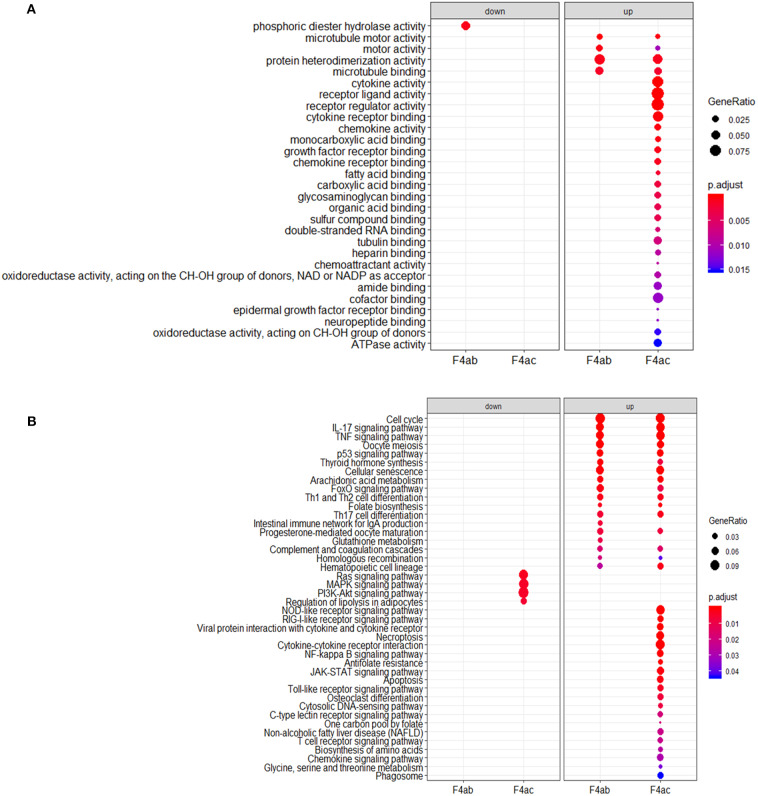
**(A)** Gene ontology and **(B)** KEGG pathway analyses of the differentially expressed genes in ETEC F4ab/ac infected cells vs. uninfected cells.

**Figure 7:**
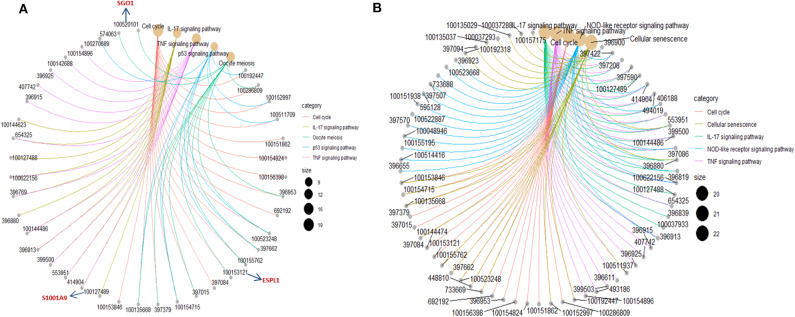
Hub pathways and associated genes in **(A)** ETEC F4ab and **(B)** ETEC F4ac infections.

To investigate the biological significance of the differentially expressed genes, we performed functional annotation analysis by using a Bioconductor bioinformatics resource. The enriched GO terms could be roughly grouped into two clusters (Figure 6A). The first cluster is factor activity, such as receptor ligand activity, receptor regulator activity, cytokine activity, and chemokine activity. The second cluster centers on receptor binding, e.g., cytokine receptor binding, growth factor receptor binding, chemokine receptor binding, microtubule binding, fatty acid binding, double-stranded RNA binding, and cofactor binding. Since a key step in ETEC infection of pigs is its binding to receptor(s) on enterocytes (Roubos-van et al., 2017), the receptor binding related genes we identified are likely to be involved in this process. KEGG pathway analysis revealed that genes were significantly enriched in some immune/defense response-related pathways (Figure 6B), e.g., IL-17 signaling pathway, TNF signaling pathway and p53 signaling pathway, which indicate their roles in response to the cytotoxic effects of ETEC. Hub nodes have been found to play important roles in networks (He and Zhang, 2006), several hub pathways and associated genes were identified for ETEC F4ab and F4ac infection independently (Figure 7). The genes induced by F4ab and F4ac share most hub pathways, which is consistent with the similarity of the F4ab and F4ac fimbrial antigens. Both have the “a” epitopes formed by the conserved region of the major F4 fimbrial subunit FaeG (Verdonck et al., 2004).

### Integrative Analysis of DNA Methylation and Gene Expression

**Figure 8:**
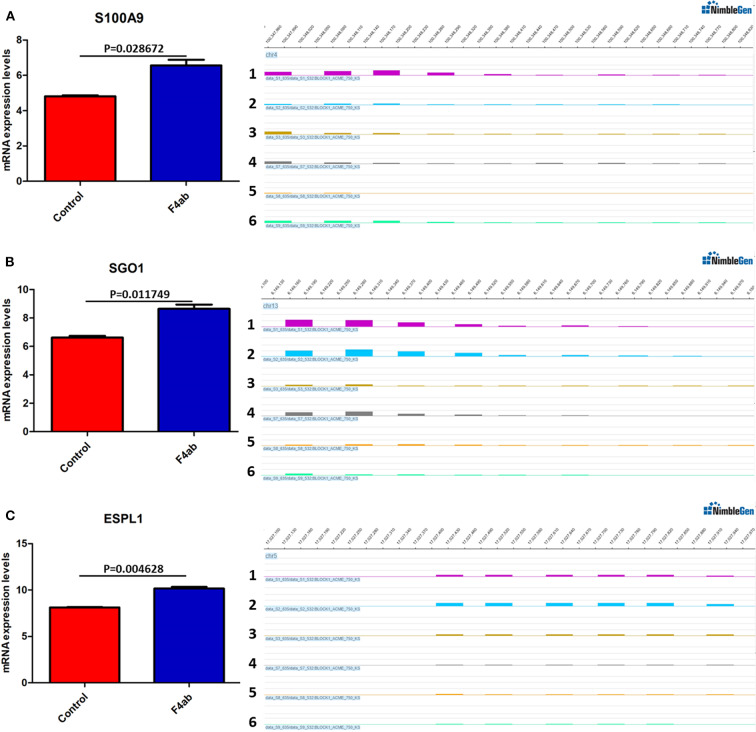
DNA methylation and expression of **(A)** *S100A9*, **(B)** *SGO1*, and **(C)** *ESPL1* in ETEC F4ab infected cells. These genes were all up-regulated (left panel) and hypomethylated (right panel) compared to uninfected controls. Panels 1-3 and 4-6 indicate the differentially methylated peaks in control and F4ab infected cells by MeDIP-chip (SignalMap software, NimbleGen). *P* < 0.01 and *P* < 0.05 indicate highly significant difference and significant difference between uninfected and F4ab infected cells, respectively.

**Figure 9:**
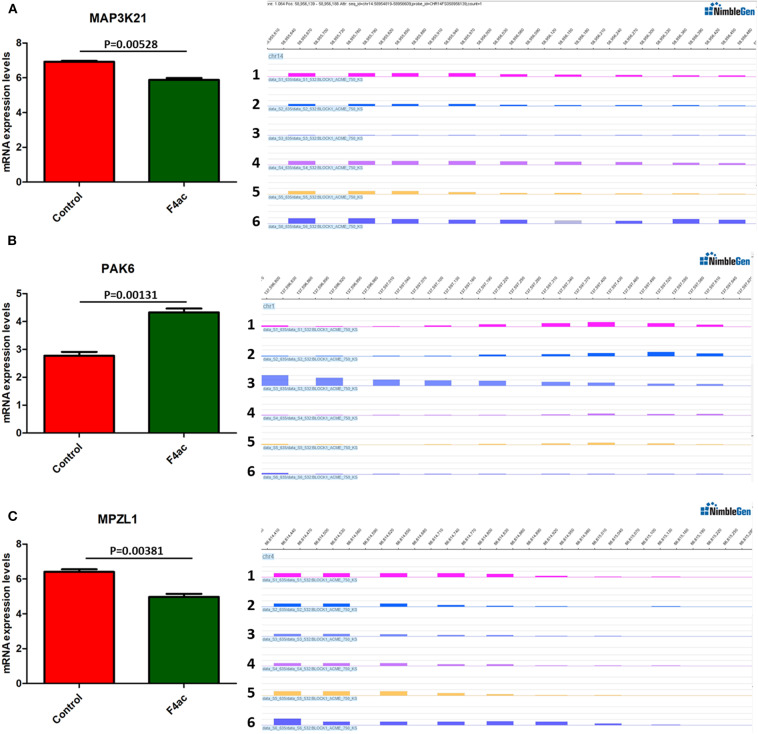
DNA methylation and expression of *MAP3K21, PAK6*, and *MPZL1* in ETEC F4ac infected cells compared to uninfected cells. **(A)** *MAP3K21* was down-regulated and hypermethylated. **(B)** *PAK6* was up-regulated and hypomethylated. **(C)** *MPZL1* was down-regulated and hypermethylated. Panels 1–3 and 4–6 indicate the differentially methylated peaks in control and F4ab infected cells by MeDIP-chip. *P* < 0.01 and *P* < 0.05 indicate highly significant difference and significant difference between uninfected and F4ab infected cells, respectively.

DNA methylation occurring at gene promoters is usually involved in inhibiting the expression of the corresponding genes (Jones, 2012). By using integrative analyses of the DNA methylation profiles and gene expression profiles from IPEC-J2 cells infected with ETEC F4ab/ac vs. uninfected cells, we explored the relationship between methylation changes at the promoter regions and gene expression changes. The results showed that among the differentially methylated genes in cells infected with ETEC F4ab and F4ac, 27 and 10 were inversely expressed with regard to their methylation status, respectively (Tables 1, 2). Genes including *S100A9, SGO1*, and *ESPL1*, which are essential for cells' antiviral response, were all hypomethylated and up-regulated in cells infected with ETEC F4ab (Figure 8), these genes were also detected in hub pathways of our transcriptome analysis (Figure 7A). The methylation and expression of *MAP3K21, PAK6*, and *MPZL1*, which play roles in immune response and adhesion, were negative correlated in ETEC F4ac infected cells *P* < 0.05 (Figure 9). Thus, these genes could be powerful candidates methylation target genes related to susceptibility or resistance to ETEC F4ab/ac.

**Table 1:** List of differentially methylated and expressed genes in ETEC F4ab infected IPEC-J2 cells.

**EntrezGeneID**	**Gene_symbol**	**Expression change**	**Methylation change**
100157217	UROC1	Upregulated	Hypomethylated
100153892	RREB1	Downregulated	Hypermethylated
100155831	CNNM2	Downregulated	Hypermethylated
100170126	LEF1	Upregulated	Hypomethylated
100147710	MEST	Upregulated	hypomethylated
100522785	E2F8	Upregulated	Hypomethylated
100127489	S100A9	Upregulated	Hypomethylated
100526242	LEPREL4	Upregulated	Hypomethylated
100511038	FKBP10	Upregulated	Hypomethylated
100737768	HBA	Upregulated	Hypomethylated
100520101	SGO1	Upregulated	Hypomethylated
100514852	STXBP6	Downregulated	Hypermethylated
733697	AK5	Upregulated	Hypomethylated
100522116	KIF23	Upregulated	Hypomethylated
100512986	ARPP21	Upregulated	Hypomethylated
100302021	CLDN8	Upregulated	Hypomethylated
100521557	C12ORF12	Upregulated	Hypomethylated
397251	PLK2	Upregulated	Hypomethylated
100524433	RECQL4	Upregulated	Hypomethylated
100153121	ESPL1	Upregulated	Hypomethylated
100518254	RPS19	Upregulated	Hypomethylated
733650	TAP2	Upregulated	Hypomethylated
100145892	DLK2	Upregulated	Hypomethylated
397544	RGS16	Upregulated	Hypomethylated
100157774	SLC25A27	Downregulated	Hypermethylated
100157115	RTDR1	Upregulated	Hypomethylated
397589	CDKN3	Upregulated	Hypomethylated

**Table 2:** List of differentially methylated and expressed genes in ETEC F4ac infected IPEC-J2 cells.

**EntrezGeneID**	**Gene_symbol**	**Expression change**	**Methylation change**
100524618	GPT	Upregulated	Hypomethylated
100155831	CNNM2	Downregulated	Hypermethylated
100155615	AVPI1	Downregulated	Hypermethylated
733644	RHCG	Downregulated	Hypermethylated
733643	NCOR2	Downregulated	Hypermethylated
100152590	NUF2	Upregulated	Hypomethylated
100627802	MTNR1A	Upregulated	Hypomethylated
100154002	MAP3K21	Downregulated	Hypermethylated
100153981	PAK6	Upregulated	Hypomethylated
100153553	MPZL1	Downregulated	Hypermethylated

## Discussion

From a genome-wide comparative methylome analysis, we revealed the epigenetic alterations in IPEC-J2 cells due to infection by ETEC F4ab/ac. We conducted an integrated analysis of MeDIP-chip and microarray data and identified a subset of genes that are implicated in the host response to ETEC F4ab/ac infection. However, these methods may only provide limited insights into the biological mechanisms of diarrhea induced by ETEC F4ab/ac. DNA, RNA, protein, and metabolite often have complementary roles to jointly perform a certain biological function. Such complementary effects and synergistic interactions between omics layers can only be captured by integrative study of multiple molecular layers (Sun and Hu, 2016). Therefore, multi-omics approaches that integrate data obtained from different omics levels (e.g., genetics, epigenetics, mRNA transcripts, proteins and metabolites) over the course of infection need to be conducted to understand their interrelation and combined influence on the host response to ETEC F4ab/ac infection. The IPEC-J2 DNA methylome profile provides an epigenetic overview of the physiological system in response to ETEC F4ab/ac infection in this study, and we expect it to constitute a set of resources for further epigenomic studies.

The host's gene expression programs especially those linked to host defense genes undergo massive changes during pathogenic infection (Boldrick et al., 2002). The epigenetic modulations such as DNA methylation can be manipulated by pathogens to influence the host's gene expression programs (Paschos and Allday, 2010). Among different kinds of epigenetic markers, DNA methylation is characterized as the most stable and easily accessible biomarker candidate. Our results provided evidences that ETEC F4ab/ac infection can trigger changes in DNA methylation and alter the expression of immune responses related genes (Tables 1, 2). In addition, other regulators such as transcription factors are also involved in the regulation of gene expression during pathogenic infection, which may in part account for the small number of common differentially methylated and expressed genes identified in our study.

Promoter methylation is directly related to transcriptional repression (Koga et al., 2009). We analyzed the methylation patterns in the promoter regions by using a porcine MeDIP-chip. In general, most chromosomal regions were covered by methylated peaks, while the methylation densities were distinct among the chromosomes. Chromosome 13 in particular, contained a relatively large unmethylated region at the end of chromosome (Figure 1), indicating that this region is highly conserved. We can't explain this phenomenon and this region need to be examined carefully in the future. The methylation levels of HCPs were higher than those of ICPs and LCPs in all IPEC-J2 cell groups (Figure 2C), which is consistent with the findings from other species (Huang et al., 2014; Su et al., 2014). The HCPs also have more methylated EPs in their promoter CGIs (Figure 2D). Koga et al. reported a stronger correlation between DNA methylation and gene repression in HCPs compared with ICPs and LCPs (Koga et al., 2009). These results suggest that promoter methylation may be correlated with pathogenic infection. Analysis of the differentially methylated genes in ETEC F4ab/ac infected cells revealed that among the 406 differentially methylated genes common to F4ab and F4ac infected cells, the majority had consistent methylation direction (Figure 4C), which is accordant with the similarity of the antigenic structures of the F4ab and F4ac fimbrial antigen (Verdonck et al., 2004). There was however a small minority of differentially methylated genes with opposite methylation direction (Figure 4C), illustrating the different methylation patterns induced by each F4 subtype.

The genes modified by differential DNA methylation merit greater attention. By integrating DNA methylation data and gene expression data, we identified 27 genes for which methylation of their promoters was inversely related to transcriptional repression in ETEC F4ab infected cells (Table 1). Among these genes, *S100A9* participates in innate immunity and mediates the inflammatory response during infection-induced inflammation (Ometto et al., 2017). In addition, Wang et al. have reported that the *S100A8/A9* recombinant attenuates bacterial adherence and invasion (Wang et al., 2018). Transfection of epithelial cells with *S100A8/A9* expression vectors increases the cells resistance to invasion by *Listeria* and *Salmonella* (Zou et al., 2013). Purified S100A8/A9 has been shown to inhibit the growth of multiple species *in vitro*, including *Escherichia coli, Candida albicans, S. aureus, K. pneumonia, Salmonella typhimurium*, and *Listeria monocytogenes* (Wang et al., 2018). Also, it has been reported that the expression of *S100A9* was actually controlled by the methylation status of its promoter (Chandra et al., 2018). SGO-1, a component of the cohesion complex, is involved in cell cycle progression, cell senescence, and activation of TGF-β signaling (Chetaille et al., 2014). Mice heterozygous for *SGO-1* showed increased chromosome instability and susceptibility to tumors (Yamada et al., 2012), and mutations in human *SGO-1* have been associated with gastric and colorectal cancers (Kim et al., 2013), as well as altered heart and gut rhythm (Chetaille et al., 2014). *ESPL1*, also known as separase, is an important regulator of the cell cycle and a potential oncogene (Kumar, 2017). For example, a homozygous mutant of *ESPL1* leads to a high level of aneuploidy thus acting as a tumor suppressor (Shepard et al., 2007). In IPEC-J2 cells infected with ETEC F4ab, these genes were all hypomethylated and up-regulated (Figure 8). They were also all detected in hub pathways of transcriptome analysis (Figure 7A). Hub nodes play important roles in networks (He and Zhang, 2006). We speculate that these genes may be functionally linked and are regulated by methylation of their promoters in response to F4ab-induced effects.

We also identified 10 genes in ETEC F4ac infected cells that had an inverse relationship between promoter methylation and gene expression (Table 2). Among these genes, *MAP3K21, PAK6*, and *MPZL1* (Figure 9) may be associated with the transcriptional repression of DNA methylation by blocking the binding of transcriptional activators or recruiting co-repressors (Klose and Bird, 2006). *MAP3K21* is a negative regulator of TLR4 signaling (Seit-Nebi et al., 2012). Toll-like receptors (TLRs) are innate immune sensors, each responding to specific molecules of microbial origin (Janeway and Medzhitov, 2002; Akira and Takeda, 2004). Binding of pathogen-associated pattern molecules, such as lipopolysaccharides (LPS), to cell surface TLRs, results in recruitment of signaling adaptors (Hoebe et al., 2003; Yamamoto et al., 2003; Beutler, 2004; Biswas et al., 2007). *MAP3K21* can suppress LPS-induced activation of the JNK or ERK pathways, but does not have an effect on LPS-induced p38 or NF-κB activation (Seit-Nebi et al., 2012). Therefore, the down-regulation and hypermethylation of *MAP3K21* after ETEC F4ac infection may be to maintain balance of the inflammatory response. *PAK6* is a member of the p21-activated kinases (PAKs) that have fundamental roles in cellular processes such as adhesion, motility, and survival, as well as in cancer progression (Field and Manser, 2012; King et al., 2014; Morse et al., 2016). MPZL1, also known as PZR, is a cell surface transmembrane glycoprotein belonging to the immunoglobulin family. It is comprised of an extracellular receptor domain and an intracellular domain with two immunoreceptor tyrosine-based inhibitory motifs (ITIMs) (Zhao and Zhao, 2003; Kusano et al., 2008). The phosphorylated ITIM motifs specifically bind to tyrosine phosphatase SHP-2, which plays an important role in cell growth factor signaling, and regulating basic cellular functions such as adhesion, proliferation, differentiation, transformation and migration (Billadeau and Leibson, 2002; Gibbins, 2002). Notably, *MPZL1* is involved in the regulation of integrin-mediated cell motility (Zannettino et al., 2003), and ETEC F4ac receptor is a member of the integrin family (Wang W. et al., 2019). Considering the functions of these genes, we believe that *MAP3K21, PAK6*, and *MPZL1*are strongly correlated with diarrhea induced by ETEC F4ac infection. It is noteworthy that only one gene, *CNNM2*, was identified to be differentially methylated and expressed in both F4ab and F4ac infected cells (Tables 1, 2). *CNNM2*, which belongs to the Cyclin M family, is an essential gene for magnesium (Mg^2+^) homeostasis (de Baaij, 2015). Mg^2+^ is a vital cofactor for more than 600 enzymes, and plays an important role in anti-inflammatory and immunomodulatory (de Baaij et al., 2015). The changes in its promoter methylation and gene expression indicate that *CNNM2* may participate in immune response to ETEC F4ab/ac stimulation.

In conclusion, we profiled the landscape of DNA methylation and gene expression in response to ETEC F4ab and F4ac infection by using a porcine intestinal epithelial cell line. Integrative analysis of the methylation and transcriptome data revealed a subset of genes implicated in the host response to ETEC F4ab/ac infection, and that the changes in expression of these genes may be driven by DNA methylation status. Thus, these genes are potential candidates for further research into the susceptibility or resistance to ETEC F4ab/ac. Our findings provide insight into the molecular effects of ETEC F4ab/ac infection and contribute to the continuing study of the epigenetic modifications resulting from ETEC F4ab/ac infection.

## Materials and Methods

### Cell and Bacterial Culture

IPEC-J2 cells were grown in Dulbecco's modified Eagle's medium (DMEM)/Ham's F-12 medium (1:1) (GIBCO, Invitrogen, Beijing) supplemented with 5% fetal calf serum (FCS, GIBCO, Carlsbad, CA, USA) and incubated in a humidified 5% CO_2_ atmosphere at 37°C. ETEC F4ab strain 195 (O8:K87:F4ab) and ETEC F4ac strain 200 (O149:K91:F4ac) were removed from cryo-storage and cultured in Ordinary Broth Agar at 37°C for three generations (24 h per generation). For cell infection experiments, strains were subcultured in LB medium and incubated with shaking (230 rpm) for 12 h at 37°C. Bacteria were collected by centrifuged and washed with sterile PBS (pH 7.4). Finally, the bacterial suspension was prepared with a final concentration of 1 × 10^8^ CFU/mL (Zhou et al., 2012).

### Infection of IPEC-J2 Cells and Nucleic Acid Isolation

Dose and time of infection optimizations experiments were conducted as described in our previous study (Zhou et al., 2012). Briefly, monolayers of IPEC-J2 cells prepared in 24-well cell culture plates (Corning, Lowell, MA, USA) were washed twice with PBS, then covered with 0.5 mL of DMEM per well. Bacterial suspension was added to the medium of experimental wells (MOI = 10:1) and an equal volume of PBS was aliquoted to the control wells. Each experimental treatment was conducted in triplicate. The cells were incubated for 3 h at 37°C in a 5% CO_2_/air atmosphere (Vieira et al., 2010) then collected for nucleic acid isolation.

Genomic DNA and total RNA were exacted by using the Qiagen DNeasy Tissue kit (Qiagen, Hilden, Germany) and TRIzol reagent (Thermo Fisher Scientific, Waltham, U.S.A.) according to the manufacturer's guidelines. Concentrations of DNA and RNA were quantified with Qubit Fluorometer (Thermo Scientific, U.S.A.). Nucleic acid integrity was further analyzed by 1% agarose gel electrophoresis with an Agilent 2100 Bioanalyzer (Agilent Technologies, Santa Clara, CA, USA.). For RNA samples, only those with an RNA integrity number >7.0 were retained for subsequent analysis.

### MeDIP-Chip

Genomic DNA from each sample was sonicated to produce random fragments 200 to 1000 bp in length. Methylated DNA was immunoprecipitated by using BioMag^TM^ magnetic beads (Bangs Laboratories. Inc) coupled to mouse anti-5-methylcytidine monoclonal antibody (Diagenode). The immunoprecipitated DNA was eluted from the beads and purified by phenol/chloroform extraction and ethanol precipitation. The input DNA and the immunoprecipitated DNA were labeled with Cy3- and Cy5-fluorophere, respectively, and hybridized to an Arraystar Custom Pig CpG Promoter array. The CpG array covered all known CpG islands annotated by UCSC and all Ensembl gene promoter regions (~−800 to +200 bp from transcription start sites), which totally covered ~385,000 probes. Scanning was performed with an Axon GenePix 4000B microarray scanner, following the manufacturer's guidelines detailed in the NimbleGen MeDIP-Chip protocol (NimbleGen Systems Inc., Madison, USA).

### Promoters and CGIs Classification

Promoter CGIs are defined as CGIs located in-−10 kb to + 0.5 kb around the TSS; intragenic CGIs are located in + 0.5 kb around the TSS to the TTS; the remaining CGIs are defined as intergenic CGIs. Key promoters are defined as the −800 to +200 bp regions around the TSS. The three categories of key promoters were determined as follows: HCP (high CpG density promoter) contains a 500bp interval with CpG/expected CpG above 0.6 and G+C content above 0.55; LCP (low CpG density promoter) do not contain a 500bp interval with CpG/expected CpG above 0.4; and ICP (intermediate CpG density promoter) is the remainders that do not fall into either HCP or LCP (Yu et al., 2014).

### Data Normalization and Analysis of MeDIP-Chip Data

The enrichment intensity was determined for each probe that mapped to gene promoters and CGIs in the MeDIP DNA and input DNA samples. Comparisons were represented as log_2_ ratio value, where the ratio was defined as the fluorescence signal of MeDIP DNA/the fluorescence signal of input DNA. *P*-values were calculated to assess whether intensity differences were significant. To avoid technical variability and to evaluate methylation differences between samples, the log_2_-transformed ratios were subjected to median centering, quantile normalization, and linear smoothing by using the Bioconductor packages Ringo (Toedling et al., 2007), Limma (Ritchie et al., 2015), and MEDME (Pelizzola et al., 2008). The normalized chip data was then analyzed by using a sliding-window (750 bp) peak-finding algorithm provided by NimbleScan v2.6 (Roche-NimbleGen). A one-sided Kolmogorov-Smirnov (KS) test was applied to determine whether the probes were drawn from a significantly more positive distribution of intensity ratios than those from the rest of the array. Each probe was associated with a –log_10_[*p*-value] score from the windowed KS test. If several adjacent probes were significantly above a set threshold, the region was classified as an enrichment peak (EP). NimbleScan detects peaks by searching for at least 2 probes above a –log_10_[*p*-value] minimum cutoff of 2. Peaks within 500 bp of each other are merged. The differential probe-level ratio (log_2_[MeDIP/Input]) between treated and untreated cells was used to analyze the MeDIP hybridization to identify differentially methylated regions (DMRs) as follows: (1) log_2_[ratio treatment]-log_2_[ratio control] > 0.25; (2) log_2_[ratio control] < 0.4; (3) *P*-value determined for treatment *vs* control < 0.05.

### Validation of MeDIP-Chip Data by Bisulfite Sequencing

Bisulfite sequencing PCR primers was designed by the online MethPrimer software (Li and Dahiya, 2002), which are listed in Table S8. Genomic DNA from the untreated IPEC-J2 cells, ETEC F4ac infected cells and ETEC F4ab infected cells were treated with bisulfite using the EZ DNA Methylation Kit (Zymo Research, D5006). Each experimental treatment was conducted in triplicate. PCR reactions were performed using the ZymoTaq™ PreMix (Zymo Research, E2004) following the manufacturer's protocols. The PCR products were then purified and cloned into the pMD19-T vector (TaKaRa biotechnology Co., Dalian, China). Ten positive clones for each subject were randomly selected for sequencing (Sangon, Shanhai, China). The final sequences were processed by the online software QUMA (Kumaki et al., 2008).

### Hybridization of cDNA Microarray

Detailed cDNA microarray procedures are described in our previous work (Zhou et al., 2012). In brief, total RNA was extracted from nine samples (three ETEC F4ab infected cells, three ETEC F4ac infected cells and three control cells) with TRIzol reagent (Thermo Fisher Scientific, Waltham, U.S.A.). Total RNA was amplified and labeled by using a Low Input Quick Amp Labeling Kit, One-Color (Agilent Technologies, Santa Clara, CA, USA), following the manufacturer's instructions. The labeled cRNA was purified by using a RNeasy mini Kit (QIAGEN, GmBH, Germany), then hybridized to porcine oligo microarray slides (Agilent Technologies) containing 43,603 oligonucleotide probes. The hybridized microarray slides were scanned by using an Agilent Microarray Scanner (Agilent Technologies, Santa Clara, CA, USA). Raw data were normalized by using the quantile algorithm from Gene Spring Software 11.0 (Agilent Technologies, Santa Clara, CA, USA). Differentially expressed genes (DEGs) were defined as those with a fold change (|FC|) > 1.5 and *P* < 0.05.

### Bioinformatics Analyses

Gene Ontology (GO) term analyses and Kyoto Encyclopedia of Genes and Genomes (KEGG) enrichment analyses for DEGs were performed by using the R package “clusterProfile” (Yu et al., 2012), with *p*-values calculated by using right-sided hypergeometric tests. To prevent a high false discovery rate (FDR) in multiple testing, q-values were also estimated for the FDR control (Storey, 2003). Figures were prepared by using the R package “ggplot2” (Wilkinson, 2011). RCircos (Krzywinski et al., 2009) was used to visualize the methylation maps of the nine samples.

## Data Availability Statement

The datasets generated for this study can be found in the datasets (GSE143661) for this study have now been deposited in the NCBI GEO database (https://www.ncbi.nlm.nih.gov/geo/query/acc.cgi?acc=GSE143661).

## Ethics Statement

The animal study was reviewed and approved by Institutional Animal Care and Use Ethics Committee of Shandong Agricultural University.

## Author Contributions

QZ, WW, and YY conceived this study. WW and CZ performed the experiments. QZ, YY, WW, and HT performed the data analyses and wrote the manuscript. All authors reviewed and approved the final manuscript.

## Conflict of Interest

The authors declare that the research was conducted in the absence of any commercial or financial relationships that could be construed as a potential conflict of interest.
